# The association between paralytic side and health-related quality of life in facial palsy: a cross-sectional study of the Korea National Health and Nutrition Examination Survey (2008–2012)

**DOI:** 10.1186/s12955-018-1038-0

**Published:** 2018-11-19

**Authors:** Sina Kim, Hye-Yoon Lee, Nam-Kwen Kim, Tae Han Yook, Eun-Sung Seo, Jong Uk Kim

**Affiliations:** 1 Center for Comparative Effectiveness Research & Economic Evaluation in Korean Medicine, Pusan National University, Yangsan, Gyeongnam South Korea; 2 National Clinical Research Center for Korean Medicine, Pusan National University Korean Medicine Hospital, Yangsan, South Korea; 3 Department of Korean Medicine, Pusan National University, Yangsan, Gyeongnam South Korea; 4 Department of Acupuncture & Moxibustion Medicine, Korean Medicine Hospital of Woosuk University, Jeonju, South Korea; 5 Department of Food and Nutrition, Seoul National University, Seoul, South Korea

**Keywords:** Facial nerve palsy, Quality of life, EQ-5D, Korea National Health and nutrition examination survey

## Abstract

**Background:**

Facial palsy is known to have correlations with low level of quality of life. However, little is known about the association between preference based health-related quality of life (HRQoL) and paralytic side of facial palsy.

**Methods:**

This study used Korea National Health and Nutrition Examination Survey (KNHANES, 2008–2012) data, only when the facial palsy examination had been included in the survey contents. Hierarchical regression analyses were used to obtain optimal regression coefficients in the association between paralytic side of the facial palsy and HRQoL measured by EuroQoL-5 Dimension (EQ-5D). We also analyzed the association between the deteriorated domains of EQ-5D and facial palsy in both subgroups by using multiple logistic regression models.

**Results:**

We included the data of 28,106 participants aged ≥19 years who were examined as facial palsy according to House-Brackmann score and completed EQ-5D questionnaire in KNHANES 2008–2012. The mean EQ-5D score was significantly low and percentages of deteriorated numbers in its domains were significantly high in facial palsy group.

**Conclusions:**

These results show that, after adjusting for confounding variables, left facial palsy is associated with impaired HRQoL compared with right-sided palsy. Among the domains of EQ-5D, only ‘self-care’ domain was directly affected by the disease in left facial palsy patients. These findings could be used in developing model and conducting analyses of economic evaluation about facial palsy interventions.

## Introduction

Unilateral peripheral facial nerve palsy may have a detectable cause (i.e. secondary facial nerve palsy) or may be idiopathic (primary) without an obvious cause (i.e. Bell’s palsy) [1, 2]. Secondary facial nerve palsy has a variety of causes and is generally less prevalent than Bell’s palsy (25 vs. 75%) [2, 3].

Bell’s palsy is an acute peripheral facial nerve paralysis that usually affects only one side of the face [4]. The clinical picture varies, depending on the location of the lesion of the facial nerve along its course to the muscles. Symptoms and signs result from the fact that the facial nerve not only carries motor fibers but also supplies autonomic innervation of the lacrimal gland, submandibular gland, sensation to part of the ear, and taste to the anterior two thirds of the tongue via the chorda tympani [5]. Thus, Bell’s palsy is diagnosed upon abrupt onset of impaired facial expression due to unilateral facial weakness, dry eye, saliva running out of the mouth, the inability to close the eye or mouth, drooping the brow or the corner of the mouth, numbness or pain around the ear, temple, mastoid, or angle of the mandible, an altered sense of taste, hypersensitivity to sounds, or decreased tearing [5, 6]. Impaired speech and difficulty in eating or drinking may also occur. Severe pain suggests herpes zoster virus and may precede a vesicular eruption and progression to Ramsay Hunt syndrome [7].

Nevertheless, all these functional discomforts, the consequences of facial nerve palsy are both functional and psychosocial, making assessment of both aspects essential to comprehensive care of the facial nerve palsy patient. The rates of anxiety and depression are much higher in patients with facial nerve palsy [8]. Patients with long-term facial nerve paralysis were reported to have lower social functioning as well [9]. The loss of self-confidence, phobic avoidance and the physical complications of paralysis lead to a significant impact on quality of life [10]. Patients require objective examination of facial movement, and subjective evaluation of the influence of facial nerve palsy on quality of life.

While most attention to health-related quality of life (HRQoL) is paid to the subgroup of facial nerve palsy patients with cerebellopontine angle pathology [11–13], the general HRQoL of patients with peripheral facial nerve palsy is not well described in the current literature. One recent study reported HRQoL of peripheral facial nerve palsy patients using the Facial Clinimetric Evaluation (FaCE) Scale [14], but the research is limited to outpatients and none of the studies have used representative population data. Thus, the aim of this study was to assess the association of facial nerve palsy and HRQoL using representative Korean data taken from the Korea National Health and Nutrition Examination Survey (KNHANES).

Moreover, we will look into the association between HRQoL and paralytic side of facial palsy. There has been a controversy whether quality of life differs according to which facial part is paralyzed. In a previous study using facial distress index and SF-36, patients with right-sided paralysis were shown to have worse psychological mood and social interactions than those with left-sided facial paralysis [15]. However, preference based instrument for assessing HRQoL is needed for developing further researches such as economic evaluation, which there has been none in this subject. Therefore, we conducted this study to estimate the preference based HRQoL of facial palsy on right and left side each with normal population data, and to ascertain which domains of EuroQoL-5 Dimension (EQ-5D) were affected by the disease.

## Materials and methods

### Study population

The KNHANES is a national surveillance system used by the Korea Centres for Disease Control and Prevention (KCDC) to assess the general health and nutritional status of Koreans. To select a representative sample of the Korean population, they used a stratified three-stage clustered probability design (local district → enumeration district → household). To date, six surveys have been completed as follows: I (1998), II (2001), III (2005), IV (2007–2009), V (2010–2012) and VI (2013–2015). Among them, data from surveys which were conducted between 2008 and 2012 contained ear, nose, and throat (ENT) examination results, as well as facial palsy grading test. Therefore, data on ENT examination results from 2008 to 2012 in the KNHANES IV and V were utilized.

**Fig. 1:**
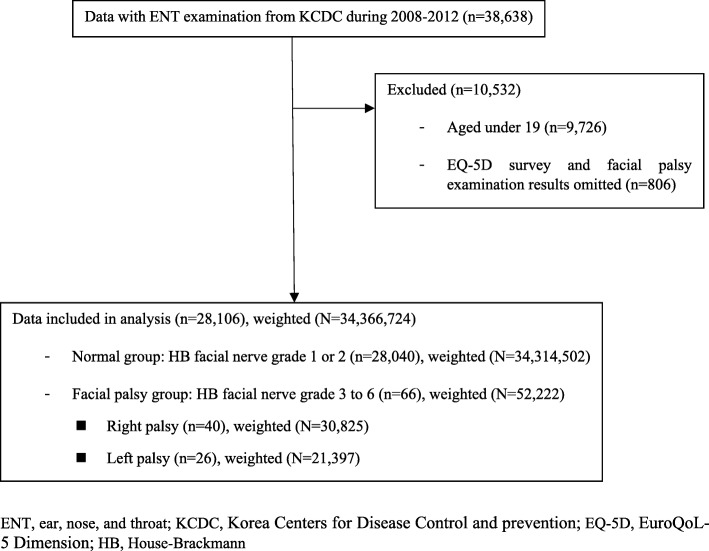
Flow diagram of the study

This study involved data from a total number of 38,638 patients. we excluded the data aged under 19 (*n* = 9726) and the data whose EQ-5D survey and facial palsy examination results were missing (*n* = 806). Finally, data from 28,106 patients were used in the analysis. A flow diagram is shown in Fig. 1.

### Evaluation of the prevalence of facial nerve palsy

Individuals who were diagnosed as grade III to VI in House-Brackmann facial nerve grading scale under ENT examination were classified as having facial nerve palsy. House-Brackmann facial nerve grading scale has been commonly used to assess recovery after trauma to the facial nerve palsy [16]. The House-Brackmann facial nerve grading scale [17] is described as follows: grade I (normal), grade II (mild dysfunction), grade III (moderate dysfunction), grade IV (moderately severe dysfunction), grade V (severe dysfunction) and grade VI (total paralysis). As follow-up showed that in 85% of patients function was naturally returned to grade I to II [3], grade I or II was regarded as full recovery and grade III to VI was classified as having facial nerve palsy. The number of patients whose House-Brackmann facial nerve grade was I or II was 28,040. These patients represented normal individuals without facial palsy. The number of patients whose House-Brackmann facial nerve grade was between III and VI was 66, and these patients were categorized as having facial palsy. Among the 66 facial palsy patients, 40 had right palsy while 26 had left palsy (Fig. 1).

### Estimation of preference based HRQoL

The EuroQoL-5 Dimension (EQ-5D) descriptive system was used to evaluate HRQoL. The EQ-5D descriptive system describes general health in terms of five dimensions: mobility, self-care, usual activities, pain/discomfort, and anxiety/depression. Each dimension has three levels, corresponding to no problems, some or moderate problems, and extreme problems, resulting in a total of 243 unique health states [18]. The EQ-5D is a well-established and internationally used generic preference based instrument for assessing HRQoL [19]. The validity and reliability of Korean EQ-5D has been previously established [20].

### Statistical analysis

Statistical analyses were performed with Stata (version 14.2, MP). KNHANES is a national-level sample survey that applies complex design (stratification, clustering, and unequal selection probabilities). Therefore, we analyzed data using stratified, cluster, and weighted variables and survey module (i.e. SVYSET) procedures. The baseline demographic characteristics of the study participants were expressed as either means (SE) or frequencies (%), as appropriate, for total participants as well as the cases with and without facial palsy. Characteristics differences by facial palsy were assessed by Rao-Scott modified chi-square test or t-test using survey procedures. Hierarchical regression analysis was used to evaluate the association between facial palsy and deteriorated of HRQoL, and regression coefficient and 95% confidence intervals were calculated after adjusting for potential confounders. Unadjusted analysis was displayed in the model 1. Socio-demographic factors, such as age, gender, income, education levels were adjusted in the model 2. Model 3 was adjusted for variables used in model 2 plus health behavior factors of smoking, drinking, and exercise. Model 4 was adjusted for the variables adjusted in model 2 plus the comorbidity of chronic disease. Moreover, for analyzing which domain of EQ-5D was directly affected in right and left facial palsy patients, we dichotomized every 3 level domain values as categorical (normal/abnormal) and conducted multiple logistic regression by adjusting all confounders used in model 4 of above multiple regression. All reported *p*-values are two-tailed, and *p* < 0.05 was considered to be statistically significant.

### Ethics statement

The survey of KNHANES phase IV and V were approved by KCDC Research Ethics Review Committee (Approval No. 2008-04EXP-01-C, 2009-01CON-03-2C, 2010-02CON-21-C, 2011-02CON-06-C, 2012-01EXP-01-2C). The need for informed consent of participants was waived by the board and research processes were conducted based on domestic & international regulations and guidelines such as the Declaration of Helsinki and the Bioethics and Safety Act.

## Results

**Fig. 2:**
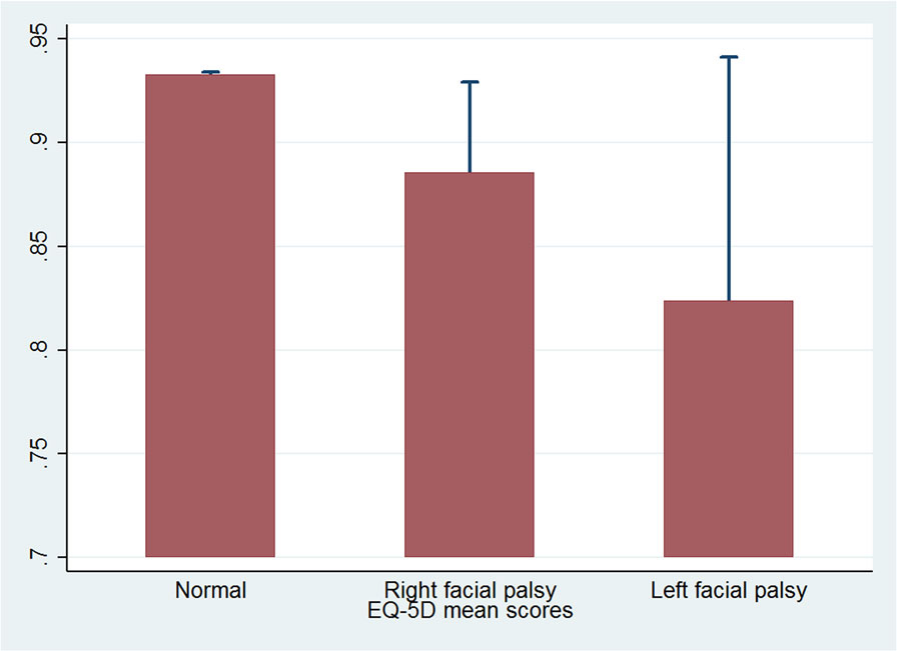
Unadjusted means and standard deviations of EQ-5D among normal, left and right facial palsy patients (*N* = 28,106)

Twenty-eight thousand one hundred six participants (sum of weights: 34,366,724), who were examined as facial palsy according to House-Brackmann score and completed EQ-5D questionnaire in KNHANES 2008–2012, were included in the study. Their socio-demographic, health behavioral, clinical characteristics are described in Table 1. In the univariate analysis, age, sex, education, house income, drinking, hypertension, diabetes, and other chronic disease were associated with right and left facial palsy prevalence. Meanwhile, variables such as smoking, stress, and cancer were not (Table 1). Facial palsy patients tended to have lower EQ-5D mean scores than normal individuals, and left facial palsy patients showed much lower scores than right facial palsy patients (Fig. 2).

**Table 1:** Characteristics of participants aged 19 and over of KNHANES, 2008–2012

Characteristics	Normal *n* = 28,040^a^ *N* = 34,314,502^b^		Right palsy *n* = 40 *N* = 30,825		Left palsy *n* = 26 *N* = 21,397		*P*-value‡
EQ-5D (mean, SE)	0.948	(0.001)	0.905	(0.024)	0.820	(0.051)	0.008
Age, year (mean, SE)	45.562	(0.201)	61.515	(2.516)	61.753	(2.611)	< 0.001
Sex, female %	0.505	(0.003)	0.797	(0.069)	0.516	(0.111)	0.005
Education, %	0.193	(0.005)	0.491	(0.097)	0.527	(0.133)	
Elementary school	0.101	(0.003)	0.172	(0.067)	0.118	(0.095)	< 0.001
Middle school	0.386	(0.005)	0.191	(0.073)	0.199	(0.110)	
High school	0.320	(0.006)	0.146	(0.073)	0.156	(0.093)	
College	0.159	(0.004)	0.302	(0.090)	0.518	(0.119)	
House income %	0.262	(0.006)	0.260	(0.093)	0.286	(0.104)	
Lowest quartile	0.295	(0.005)	0.230	(0.084)	0.105	(0.072)	0.001
Lower quartile	0.284	(0.006)	0.209	(0.081)	0.091	(0.078)	
Higher quartile	0.252	(0.004)	0.076	(0.052)	0.219	(0.102)	
Highest quartile	0.574	(0.005)	0.288	(0.088)	0.526	(0.112)	
Smoking %	0.280	(0.004)	0.287	(0.096)	0.357	(0.101)	0.123
Drinking %	0.178	(0.004)	0.449	(0.097)	0.635	(0.119)	0.010
Stress %	0.066	(0.002)	0.210	(0.089)	0.093	(0.070)	0.749
Hypertension %	0.057	(0.002)	0.018	(0.018)	0.148	(0.103)	< 0.001
Diabetes %	0.486	(0.005)	0.651	(0.094)	0.857	(0.075)	0.021
Cancer %	0.948	(0.001)	0.905	(0.024)	0.820	(0.051)	0.179
Other Chronic disease %	45.562	(0.201)	61.515	(2.516)	61.753	(2.611)	0.001

Data are presented as the mean (standard error), or % (standard error). Smoking, drinking, stress variables are dichotomous of current state

^a^Participant number of KNHANES

^b^Weighted number of the Korean population aged ≥19

‡ *P*-values from t-test or Rao-Scott chi-square test for continuous or categorical variables

**Fig. 3:**
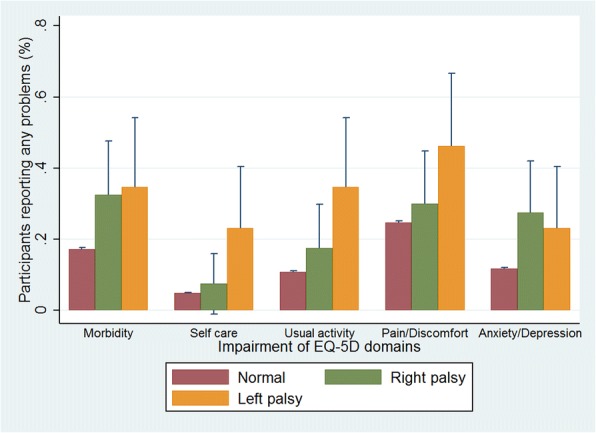
Unadjusted impaired percent (normal/abnormal) and standard deviations of EQ-5D domains among normal, left and right facial palsy patients (*N* = 28,106)

Hierarchical regression analysis was used to evaluate the association between facial palsy and deterioration of HRQoL (Table 2). In an unadjusted analysis (model 1), both right and left-sided facial palsy patients showed significantly worse conditions in quality of life. But when confounding variables were adjusted in model 2–4, there was no significant difference in quality of life among right facial palsy patients, whereas the difference still existed in left facial palsy patients. Similarly, in an unadjusted analysis in Fig. 3, facial palsy patients tended to report more problems in all five EQ-5D domains than normal individuals, but the left facial palsy patients showed higher frequency of problems than right facial palsy patients in four domains except ‘anxiety/depression’ domain.

**Table 2:** Association between right, left facial palsy and preference based quality of life measured by EQ-5D in participants aged 19 and over of KNHANES, 2008–2012

Dependent variable	Rt. Facial palsy			Lt. facial palsy		
EQ-5D	β	SE	*p*-value	β	SE	*p*-value
Model 1	−0.047	0.020	0.021	−0.109	0.025	< 0.000
Model 2	0.004	0.019	0.841	−0.052	0.022	0.021
Model 3	0.004	0.018	0.824	−0.052	0.022	0.019
Model 4	0.004	0.018	0.830	−0.049	0.022	0.028

Model 1, no adjustment; Model 2, adjusted for sex and age; Model 3, adjusted for sex, age, household income, education level, smoking, and drinking; Model 4, adjusted for sex, age, household income, education level, smoking, drinking, hypertension, diabetes, and other chronic disease (arthritis, heart disease, liver and kidney disease)

*β* regression coefficient, *SE* standard error

Multiple logistic regression (multivariable-adjusted odds ratios) was conducted to analyze the association between facial palsy and deterioration of five domains of EQ-5D (Table 3). Right facial palsy patients showed no statistical significance in any of the five domains, whereas left facial palsy patients showed declined ability in self-care.

**Table 3:** Multivariable-adjusted odds ratios for the impaired domains of the EQ-5D scores of left and right facial palsy

Dependent variable	Rt. Facial palsy			Lt. facial palsy		
EQ-5D domains	OR	95% CI	*p*-value	OR	95% CI	*p*-value
Mobility	1.168	(0.542, 2.513)	0.692	0.940	(0.386, 2.289)	0.892
Self-care	0.822	(0.243, 2.782)	0.753	2.860	(1.076, 7.600)	0.035
Usual activities	0.893	(0.369, 2.163)	0.802	1.900	(0.786, 4.595)	0.154
Pain/discomfort	0.637	(0.299, 1.358)	0.243	1.424	(0.630, 3.219)	0.396
Anxiety/depression	2.063	(0.971, 4.384)	0.060	1.422	(0.555, 3.644)	0.463

Logistic model with multiple covariates of Model 4 in Table 2 (sex, age, household income, education level, smoking, drinking, hypertension, diabetes, other chronic disease) were used for assuming OR and CI

*OR* odds ratio, *CI* confidence interval

## Discussion

To our knowledge, this is the first study to analyze the relationship between paralytic side of facial palsy and HRQoL with a representative Korean sample. Using the KNHANES database, we compared overall difference of HRQoL between facial palsy and normal individuals and then stratified by right and left paralytic sides.

Facial palsy causes difficulties in talking, eating, involuntary spasms and cosmetic deformities, which can give rise both to severe psychological and physical trauma [21]. It also affects the society’s perception of individuals by others, which could cause a devastating effect on the economic aspects of the person’s life. Therefore, all of these components could lead to the deterioration of quality of life (QoL) in facial palsy patients.

There has been studies to verify the hypothesis that the same disease could influence the QoL differently according to the affected disease side. Foster et al reported that left hemibody onset patient had lower QoL than right in Parkinson’s disease [22], and Wasan et al found that left side spinal pain had worse QoL than right side spinal pain [23]. Regarding preference side and different influences of QoL by stroke, de Haan et al used the Sickness Impact Profile to compare QoL in patients with hemiplegia, and found that there was weak relationship of more QoL deterioration in patients with right-sided lesions compared with left-sided [24]. However, Nam et al investigated whether the paralysis of dominant hand affected QoL in patients with subacute stroke and found no significant differences in QoL between the dominant hand side paralysis and non-dominant side paralysis [25].

Ryu et al studied right or left-sided facial palsy and QoL using SF-36 and reported that right-sided facial palsy showed worse psychological mood and social interaction than left-sided palsy [15]. These results were thought to be caused by more stressful situation to patients, given that the facial defect is related to emotions and unspoken communications commonly signaled through facial expressions in an affected person’s daily life. However, some controversial reports also exist that healthy individuals prefer the left to right side in relation to perceptual and attentional asymmetries of faces.

In this context, we designed this study to investigate whether right or left-sided facial palsy differently affect the preference based QoL using nation-wide survey data of KNHANES. In Korea, the survey conducted otolaryngology examinations (by specialists) including facial palsy during the period of 5 years between 2008 and 2012. Moreover, we used preference based HRQoL data measured by EQ-5D three level and estimated by validated tariff for Korean population, of which results might be useful for developing and conducting economic evaluation about the related interventions of facial palsy.

In this study, we could find that the mean EQ-5D of facial palsy was significantly lower than that of normal population without adjustment. Mean differences between normal and both (right and left) subgroups were also statistically significant (Fig. 2). After adjusting socio-demographic, health behavioral and other disease related factors, only coefficients of association in left facial palsy still remained statistically significant, but did not in right facial palsy (Table 2). The regression coefficient, which was used in the final model (Model 4 in Table 2) to predict the EQ-5D of left facial palsy, indicated a lower QoL during left facial palsy, and this result is also consistent in Table 3, which indicates that the risk of having deteriorated ‘self-care’ ability in left facial palsy group is 2.86 times higher than normal population.

These results cannot be explained by our study, but based on previous studies, possible assumptions can be made. First of all, the right hemisphere is known to be superior in recognizing emotions expressed by the face. It is also known to be specialized in processing emotional information; therefore, the left visual field (= left face reflected in a mirror), which is projected on the cortex of the right hemisphere, may be important in emotional impression [26–28]. The reading habits of Koreans, who read texts from left to right, can also explain the results [27]. People who read in this way accept information firstly from the left visual field. Patients with left facial palsy have their impaired face in the left visual field when they observe their faces in mirrors, and consequently may get relatively more negative impression. Furthermore, a study by Manovich et al. [29] demonstrated that a photo of the left side of the face resulted in a greater negative emotional expression, compared to the right side of the face. Left facial palsy patients would more likely stare at the left side of their face, which reflects a more negative expression, compared to the right side. Thus, this study could explain why patients with left facial palsy tend to have decreased satisfaction and QoL compared to those with right facial palsy. However, our results did not match Ryu’s previous report regarding that HRQoL of right palsy is worse than that of left palsy. Regarding this, many prior researches reported that different instruments could estimate HRQoL differently in many diseases, which could be considered as the cause of it. Moreover, SF-36 used in Ryu’s research is a general HRQoL measurement which is focused on health-related multi-dimensions, and EQ-5D in our research is developed for measuring preference based HRQoL, which is mainly used for calculating quality adjusted life-year gains (QALYs) and disease model development in economic evaluations. Therefore, future research should be needed for providing conclusive results about this mismatching point.

Like other researches using established national survey data, there are several limitations in our research which are as follows. Firstly, we could not include disease severity and duration of facial palsy in the model which might directly impact the HRQoL level. This is due to lack of information in the original data. However, the severity of facial palsy is known to affect the HRQoL [30]. In addition, longer disease duration, chronic pain, and impaired function (e.g. ability to smile) have been reported to influence the HRQoL [30, 31]. Thus, future studies involving these factors are required. Secondly, we could not analyze HRQoL of facial palsy using measurements other than EQ-5D, which prevented us from ascertaining the cause of discrepdPancies between the prior research result and ours. Lastly, although KNHANES is known to be designed reflecting the numbers of participants and the response rate of survey lesions for representing national population, the facial palsy prevalence might not be properly considered in the weighting and sampling process, which might disturb generalizing our research results as a national level.

In spite of above several limitations, this study is the first to examine the relationship between left, right facial palsy and preference based HRQoL using national survey data. The results of this study will contribute to offering the estimates of preference based HRQoL of facial palsy, which might be an essential component of conducting economic evaluation about the interventions of this disease. Moreover, our results also suggest that, at least in the paralyzed disease of facial area such as facial palsy, blindness, and deafness, paralyzed side could influence the preference based HRQoL of individual patients differently.

## Conclusion

We found that preference based HRQoL of left facial palsy was lower than right palsy, and overall EQ-5D score and impaired ‘self-care’ domain of EQ-5D was clearly associated with left facial palsy after adjusting sociodemographic, health behavioral and disease comorbidity conditions. Future studies that examine the relationship between HRQoL and facial palsy incorporating both general HRQoL and preference based HRQoL instruments, and other medically recorded variables including disease severity and durations are needed.

## Abbreviations

ENT: Ear, nose and throat
EQ-5D: EuroQoL-5 Dimension
FaCE: Facial Clinimetric Evaluation
HRQoL: Health-related quality of life
KCDC: Korea Centers for Disease Control and prevention
KNHANES: Korea National Health and Nutrition Examination Survey
QALYs: Quality adjusted life-year gains
QoL: Quality of life
